# Discovering multiple transcripts of human hepatocytes using massively parallel signature sequencing (MPSS)

**DOI:** 10.1186/1471-2164-8-207

**Published:** 2007-07-02

**Authors:** Jian Huang, Pei Hao, Yun-Li Zhang, Fu-Xing Deng, Qing Deng, Yi Hong, Xiao-Wo Wang, Yun Wang, Ting-Ting Li, Xue-Gong Zhang, Yi-Xue Li, Peng-Yuan Yang, Hong-Yang Wang, Ze-Guang Han

**Affiliations:** 1 Department of Chemistry of Fudan University & Shanghai-Ministry Key Laboratory of Disease and Health Genomics, Chinese National Human Genome Center at Shanghai, 351 Guo Shou-Jing Road, Shanghai 201203, China; 2 SCBIT-Inforsense Joint Lab, Shanghai Center for Bioinformation Technology, 100 Qinzhou Road, Shanghai 200235, China; 3 School of Life Science, Fudan University, 220 Handan Road, Shanghai 200433, China; 4 Shanghai Institutes for Biological Sciences, Chinese Academy of Sciences, 320 Yue Yang Road, Shanghai 200031, China; 5 International Co-operation Laboratory on Signal Transduction, Eastern Hepatobiliary Surgery Institute, the Second Military Medical University, Shanghai 200438, China; 6 MOE Key Laboratory of Bioinformatics, Department of Automation, Tsinghua University, Beijing 100084, China

## Abstract

**Background:**

The liver is the largest human internal organ – it is composed of multiple cell types and plays a vital role in fulfilling the body's metabolic needs and maintaining homeostasis. Of these cell types the hepatocytes, which account for three-quarters of the liver's volume, perform its main functions. To discover the molecular basis of hepatocyte function, we employed Massively Parallel Signature Sequencing (MPSS) to determine the transcriptomic profile of adult human hepatocytes obtained by laser capture microdissection (LCM).

**Results:**

10,279 UniGene clusters, representing 7,475 known genes, were detected in human hepatocytes. In addition, 1,819 unique MPSS signatures matching the antisense strand of 1,605 non-redundant UniGene clusters (such as *APOC1*, *APOC2*, *APOB *and *APOH*) were highly expressed in hepatocytes.

**Conclusion:**

Apart from a large number of protein-coding genes, some of the antisense transcripts expressed in hepatocytes could play important roles in transcriptional interference via a *cis*-/*trans*-regulation mechanism. Our result provided a comprehensively transcriptomic atlas of human hepatocytes using MPSS technique, which could be served as an available resource for an in-depth understanding of human liver biology and diseases.

## Background

The liver – one of most important organs in the human body – performs the main digestive function in the metabolism of most substances. In addition, liver has a number of other functions, including the generation of red blood cells during embryonic development, production of various plasma proteins, detoxification of xenobiotics and phagocytosis of solid materials. It forms a protective barrier between the digestive tract and the rest of the body. The liver also plays a vital role in activation, catabolism and excretion of retinols that are essential to the vision, growth, reproduction, immunity, cell proliferation and differentiation of the body. Furthermore, as a major organ of drug elimination, the liver has a significant effect on drug metabolism. However, to date the molecular mechanisms of liver function have not been completely characterized.

Massively Parallel Signature Sequencing (MPSS) as a global view with no bias towards the transcriptome of certain tissues or cells will provide a profound understanding of organ and cell functions [1,2]. It is well known that human liver is composed of many types of cells, such as hepatocytes, bile duct cells and kupffer cells, where hepatocytes account for three-quarters of the volume and perform the main functions of the liver. In this study, a powerful transcriptomic approach was employed to profile the gene expression of human hepatocytes obtained by laser capture micro dissection (LCM) for providing an available resource to address the molecular basis of hepatocyte biology.

## Results and Discussion

### Identification of UniGene clusters in hepatocytes by MPSS

**Figure 1:**
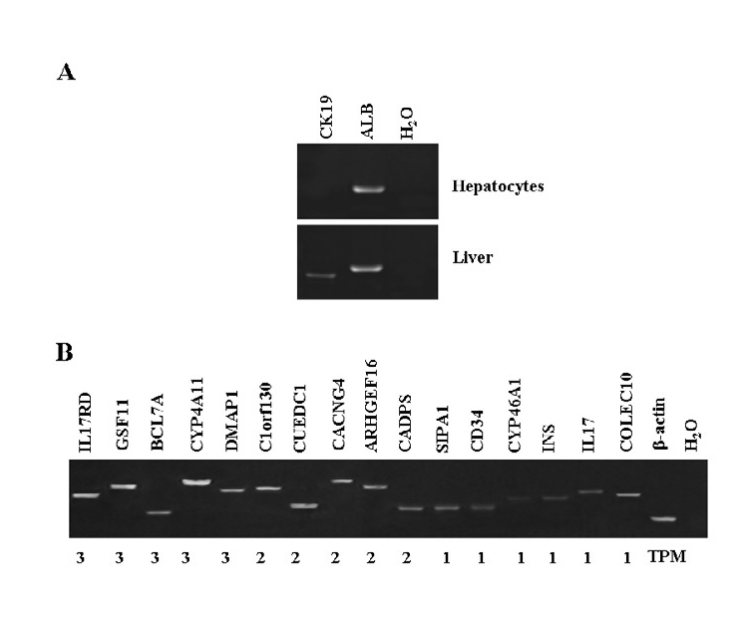
Evaluation of RNA samples from human liver and hepatocytes. **A**. RT-PCR was employed to evaluate the quality of RNA samples from human hepatocytes (right) by detecting the transcripts of albumin (ALB) and cytoskeleton protein 19 (CK19), where human liver (left) was used as reference and H_2_O was employed as control. Each PCR was performed using 35 thermal cycles and then the PCR products were observed by electrophoresis on 2% agarose gels. **B**. 16 genes with less than 3 TPM were randomly selected for the evaluation of those MPSS signatures with low frequency via RT-PCR, where β-actin and H_2_O were employed as internal and negative controls, respectively. Each PCR was generally performed using 35 thermal cycles and then the PCR products were observed by electrophoresis on 2% agarose gels. The numbers indicate the TPM of the MPSS signatures.

First, human hepatocytes were obtained from the ten samples using LCM. To eliminate the variation between individuals, we extracted the total RNA from these individual samples and assessed by the Agilent 2100 Bioanalyzer, and then RNAs were equally pooled. The pooled RNA from the LCM samples was available for analysis in following steps. To further evaluate the quality of RNA sample isolated from the hepatocytes, reverse transcription (RT)-PCR was employed to detect the expression of the hepatocyte-specific marker, albumin (ALB), and the bile duct cell-specific marker, cytoskeleton protein 19 (CK19). The resulting data showed that ALB was significantly expressed in the hepatocytes (Figure 1A, upper), whereas CK19 was detected in liver but not in the hepatocytes (Figure 1A, bottom), indicating that the hepatocytes captured by LCM were pure and appropriate for the transcriptomic analysis.

The pooled RNA was then subjected to MPSS analysis (TaKaRa Co., Japan). 60,635 distinct signatures that represent different transcripts were derived from a total of 2,990,779 sequences in four sequencing runs. The sequencing runs were merged and the expression level of each signature was normalized to transcripts per million (TPM), essentially as previously described by Meyers *et al *[1-3]. To annotate the signatures, the distinct signatures were searched against human UniGene database (UniGene Build 184, Homo sapiens) and the human genome sequences (hg17) by BLAST programs. Similar to the annotation process developed by Lynx, each signature was ranked based on its position and orientation within the transcript, the presence of a polyadenylation signal and polyA tail in the transcript sequence (see additional file 1). Herein, the expression value (TPM) of each gene is the sum of all TPMs of signatures matched to this gene. Out of the 60,635 signatures, 92.3% and 6.5% were detected in the range of 1–9 and 10–99 TPM, respectively. Only 1.2% of the signatures exhibited the high TPMs over 100 (Table 1), in which ALB (97934 TPMs), known to be secreted by hepatocytes, showed the highest abundance. Overall, the abundances of MPSS signatures indicated that a large number of genes were expressed at lower levels in hepatocytes, whereas only a few genes were expressed at very high levels.

**Table 1:** Distribution of Unique MPSS signatures with expression levels

TPM*	MPSS signatures	
	
	n	%
≥ 10000	6	0.01
1000–9999	83	0.14
100–999	640	1.06
10–99	3,958	6.53
4–9	8,191	13.51
2–3	23,028	37.98
1	24,729	40.78

total	60,635	100.00

TPM:transcripts per million

To describe the detailed profiles of gene expression of hepatocytes, 60,635 signatures from MPSS data were first compared with UniGene database. The resulting data indicated that 19,435 (32.05%) signatures were uniquely matched with 10,279 non-redundant UniGene clusters, whereas 7,223 (11.91%) signatures could not be defined as unique UniGene clusters due to multiple hits with UniGene dataset. The remaining 33,977 signatures were then employed to be searched against a human genomic database. 15,807 (26.07%) signatures were uniquely mapped onto specific genomic loci, whereas 11,614 (19.15%) hit multiple genomic sequences (Table 2). In addition, 2,229 (3.68%) signatures matched with over 100 genome locations. 4,327 (7.14%) signatures could not be matched to any known genomic sequences, possibly due to sequencing errors, spliced 3'-ends that have not yet been identified, and transcripts in regions of the genome not yet sequenced [4,5]. Alternatively, the reasons why signatures may not match the genome may be that the signatures contain part of a poly(A) tail, or polymorphisms between the individual(s) analysed and the reference human genome sequence, or contamination with RNA or DNA from other species. Also, the use of BLAST to match signatures to the genome may preclude the identification of single nucleotide mismatches between the sequence and the genome or transcriptome databases.

**Table 2:** Classification of MPSS signatures expressed in hepatocytes

Annotation	MPSS signatures		UniGene clusters
		
	n	%	
UniGene-single	19435	32.05	10279
UniGene-multi	7223	11.91	/
Genome-single	15807	26.07	/
Genome-multi	11614	19.15	/
repeat	2229	3.68	/
no hit	4327	7.14	/

Total	60635	100.00	10279

To evaluate whether those signatures with low abundance (≤ 3 TPMs) were also reliably expressed in the hepatocytes, RT-PCR was employed to detect the transcripts of the 16 UniGene clusters with less than 3 TPMs, where the UniGenes were randomly selected for the estimation. The interesting results showed that the transcripts of all UniGene clusters examined were indeed detected in the human hepatocytes at different levels (Figure 1B), suggested that transcripts detected at 3 TPMs or less by MPSS assay could in fact be proven to be expressed by RT-PCR.

Taken together, the resulting data suggested that 10,279 UniGene clusters could be detected in human hepatocytes using MPSS technique, implying that 7,475 known genes represented by the MPSS signatures could be expressed in the cell type of liver (see additional file 2).

### Enriched genes in hepatocytes

**Figure 2:**
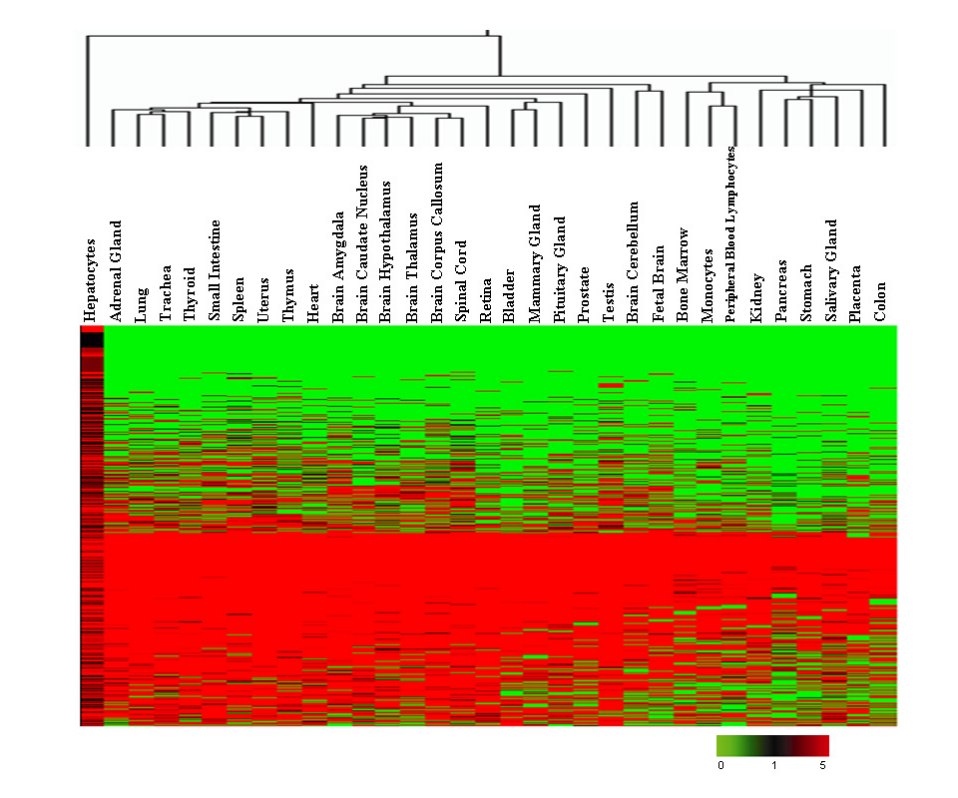
Hierarchical cluster analysis on MPSS signatures from 33 human tissues. Gene clustering was performed to analyze the expression patterns of 10,279 UniGene clusters using a log_2 _pseudocolor scale, as indicated by fold ratio in the legend below, where green and black denote 0 and 1 TPM respectively, and red indicates MPSS signatures with more than 1 TPM.

To explore the enriched genes expressed in human hepatocytes, we compared the hepatocyte MPSS dataset with datasets from another 32 tissues deposited in GEO [6]. We clustered all genes with hierarchical clustering by using Genespring Software (Figure 2). The resulting data showed that there were many genes enriched in hepatocytes. To identify the enriched genes in hepatocytes, the following formula was used as previous description [6]:

S=log⁡(Eh+1∑t=1nEt−Eh+1)2
 MathType@MTEF@5@5@+=feaafiart1ev1aaatCvAUfKttLearuWrP9MDH5MBPbIqV92AaeXatLxBI9gBaebbnrfifHhDYfgasaacH8akY=wiFfYdH8Gipec8Eeeu0xXdbba9frFj0=OqFfea0dXdd9vqai=hGuQ8kuc9pgc9s8qqaq=dirpe0xb9q8qiLsFr0=vr0=vr0dc8meaabaqaciaacaGaaeqabaqabeGadaaakeaacqWGtbWucqGH9aqpcyGGSbaBcqGGVbWBcqGGNbWzdaWgbaWcbaGaeeOmaidabeaakiabcIcaOmaalaaabaGaemyrauKaemiAaGMaey4kaSIaeGymaedabaWaaabCaeaacqWGfbqrcqWG0baDcqGHsislcqWGfbqrcqWGObaAcqGHRaWkcqaIXaqmaSqaaiabdsha0jabg2da9iabigdaXaqaaiabd6gaUbqdcqGHris5aaaakiabcMcaPaaa@48E8@

Here *S *is the enrichment, *E1 *to *En *are the expression levels across all tissues and *Eh *is the expression value observed in hepatocytes for a given gene. *S *values > 2 would be considered as hepatocyte-enriched genes. The results indicated that 327 non-redundant UniGene clusters were enriched in hepatocytes (see additional file 3). Many of these were well-known to be secreted by hepatocytes and enriched in the liver. For example, *ALB*, *APOA*, *APOB*, and *APOC *are related to lipid metabolism and maintaining the balance of proteins in plasma. Interestingly, a number of genes with unknown functions, such as *FLJ32745 *and *MGC40405*, were also enriched in hepatocytes. Whether these genes are involved in hepatic functions should be further investigated. However, it should be pointed out that the distinct discrepancy of gene expression profiles between liver and other human tissues, based on hierarchical clustering of MPSS data (Figure 2), might result partially from the technical difference between the data collected in this study and the reference dataset.

**Figure 3:**
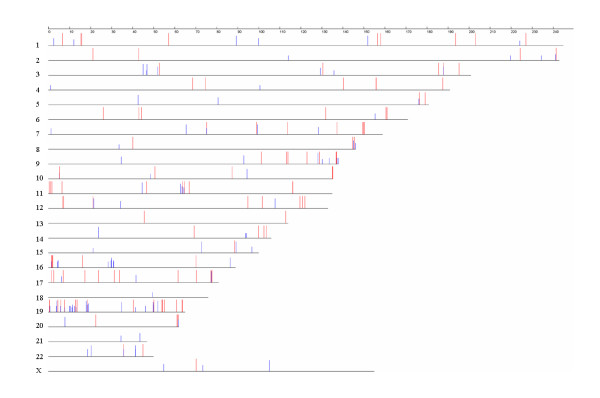
Chromosomal localization of 327 hepatocyte-enriched genes. The vertical blue bars indicate the frequency (0–10 TPMs) of the MPSS signatures of these genes, whereas the red bars represent the signatures with more than 10 TPMs.

**Figure 4:**
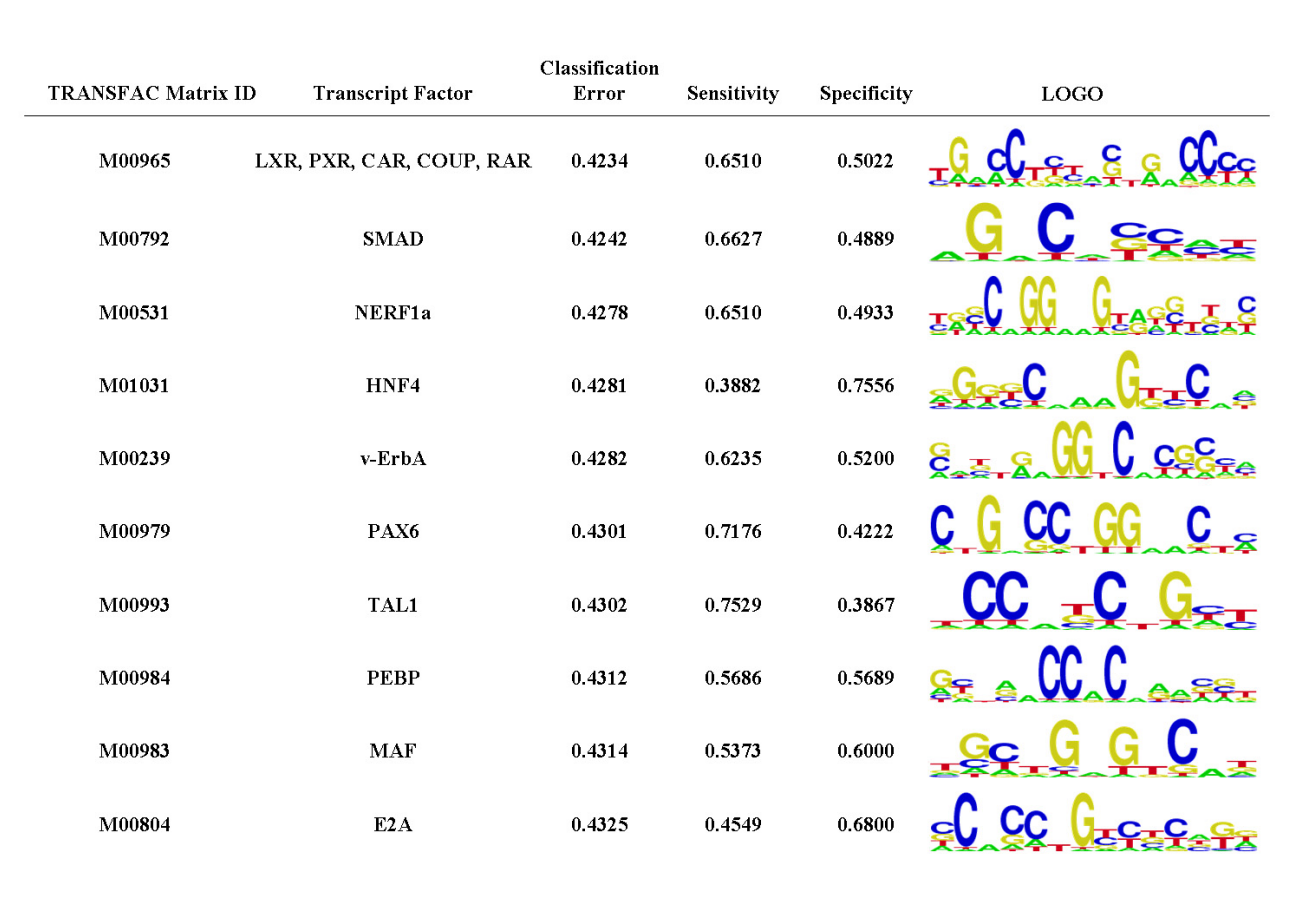
The top ten TRANSFAC motifs with the lowest classification error in the promoters of the hepatocyte-specific genes. The motifs in the promoters (-1000 bp-200 bp around the transcription start sites) of hepatocyte-specific genes (*S*_value more than 2) were compared with those of non-hepatocyte-specific (*S*_value less than -2) using Motifclass software. For each motif the TRANSFAC matrix ID, corresponding binding factors, and the classification error ratio (broken down into sensitivity and specificity) are shown in columns. Sequence logo builds from the PWM for the motif matrix enriched in hepatocyte-specific genes are shown on the right.

These 327 hepatocytes-enriched genes were mapped onto human chromosomes based on genomic information (GeneMap'99). Some hepatocyte-enriched UniGene clusters were mapped onto certain gene-rich regions, such as 8q24, 11p15.5, 11q13.1, 16p13.3, 17q25.1 and 19 (Figure 3). To further characterize the regulatory mechanisms of the genes enriched in hepatocytes, the most over-represented motifs of the promoters of these hepatocyte-enriched genes (*S*_value more than 2), as compared to that of those non-hepatocyte-enriched genes (*S*_value less than -2), were identified *in silico *by the Motifclass program [7]. The top ten over-represented motifs included NR1H3, SMAD, NERF1a, and HNF4, where the corresponding sequence logo builds were derived from the position weight matrix (PWM) for the motifs (Figure 4). Among them, HNF4 – one of the best experimentally known motifs in human liver – has been reported to play an important role in gene regulation networks in liver [8,9]. The data suggested that these *cis*-regulatory elements and their corresponding transcription factors might be crucial to the expression pattern of the hepatocyte-enriched genes.

### Antisense transcripts

Natural antisense transcripts (NATs) have been identified from plants [10] and animals [11], and these antisense RNAs are thought to be very important in the regulation of gene expression in such higher eukaryotes [11,12]. Recently, approximately 5000 UniGene clusters of potential human NAT pairs had been identified by several groups [13-15]. The synchronous presence of both sense and antisense transcripts in the same cells or tissues may be an important indicator of antisense regulation [16,17]. Interestingly, among 19,435 signatures uniquely matched with UniGene clusters, we here identified 1,819 unique signatures matching antisense strands according to the signature classification (see additional file 1 and 4), according to the previous description by Jongeneel *et al *[6]. Herein, these antisense strands represented 1,605 non-redundant *cis*-antisense transcripts in hepatocytes, of which 1,127 (70.2%) of these antisense transcripts are co-expressed with their corresponding sense transcripts, whereas 478 (29.8%) of these antisense transcripts were found to be expressed alone. Compared with published NATs, 1,222 out of 1,605 UniGene clusters of the antisense transcripts were previously uncharacterized, implying that a large number of NATs could be found in given cells or tissues by the powerful transcriptomic approach.

**Figure 5:**
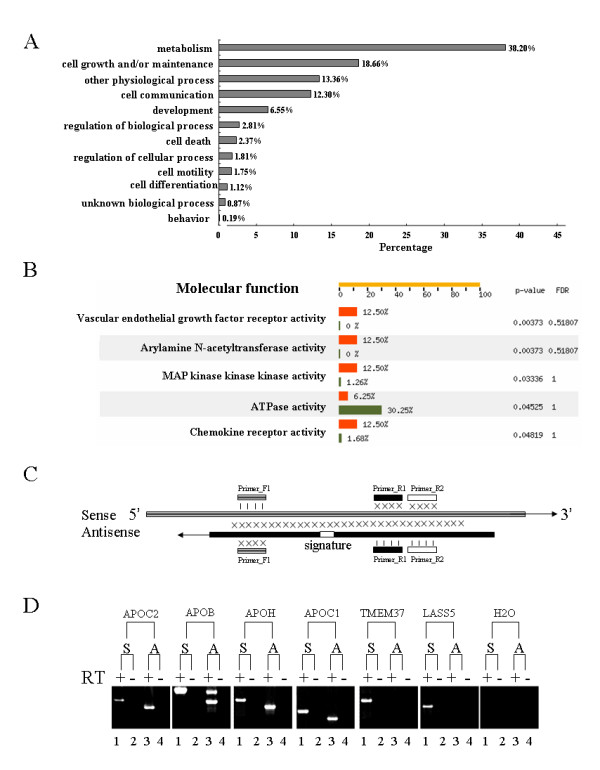
Functional categories of genes with antisense transcripts and confirmation of sense-antisense pairs of various genes expressed in human hepatocytes. **A**. Genes with antisense transcripts were assigned into different functional categories according to the Gene Ontology (GO) database. **B**. Functional over- or under-representation of GO subcategories in genes with antisense transcripts was statistically analyzed using FatiGO+ classification tool, as compared to genes without detectable antisense transcripts (p < 0.05). Red boxes indicated the GO subcategories related to genes with antisense transcripts; Green ones indicated the subcategories related to genes without detectable antisense transcripts. **C**. Sketch map of specific PCR primers designed for detecting both sense and antisense of various genes. The cross signs between antisense and sense transcripts represent complementarity each other, whereas the vertical bars indicate identical base pairs. Reverse transcription was performed using the specific primers (Primer_F1 for the antisense transcripts) that only hybridize to the sense or antisense transcripts. **D**. The sense and antisense transcripts of APOC2, APOB, APOH, APOC1 were amplified by RT-PCR using specific primers, where primers F1 and R2 were used for the sense transcripts (lane 1) and primers F1 and R1 for the antisense transcripts (lane 3). Lanes 2 and 4 were negative controls without cDNA synthesis (with DNase-treated total RNA). The two genes (TMEM37 and LASS5) without antisense counterparts were served as the set of negative control.

To further characterize the functional features of those genes with antisense transcripts expressed in human hepatocytes, these genes were assigned into GO functional categories. The majority of these genes were classified into the categories of basic metabolism, cell growth and/or maintenance, and cell communication, whereas a few genes were associated with liver development, morphogenesis, cell differentiation, and cell death (Figure 5A). To define whether a GO category is really over- or under-represented in genes with antisense transcripts, we here performed the FatiGO+ classification, a web-based tool for the functional profiling of GO categories, through comparing the subcategories for genes with or without detectable antisense transcripts. Interestingly, the resulting data suggested that some GO subcategories related to molecular functions, such as the activity of vascular endothelial growth factor receptor, arylamine N-acetyltransferase, MAP kinase kinase kinase and chemokine receptor, were significantly over-represented in genes with antisense transcripts (p < 0.05), whereas the category associated with ATPase activity were under-represented in genes with antisense transcripts (p < 0.05) (Figure 5B), suggesting that NATs could be involved in the regulation of hepatocyte functions. Both sense and antisense transcripts of some genes that are related to liver function were found to be co-expressed in hepatocytes. For example, some apolipoproteins (apo), including *APOC1*, *APOC2*, *APOB *and *APOH *that constitute the essential structural proteins of certain lipoproteins involved in lipid transport, were found to exhibit both sense and antisense transcripts co-expressed in hepatocytes. To confirm the existence of the antisense transcripts, specific primers against these genes were designed for RT-PCR (Figure 5C), where two genes, TMEM37 and LASS5, without antisense counterparts detected by MPSS, were randomly selected as negative control in the RT-PCR approach. The RT-PCR data showed that the antisense transcripts of *APOC1*, *APOC2*, *APOB *and *APOH *are indeed co-expressed with their corresponding sense transcripts in hepatocytes (Figure 5D), whereas the antisense counterparts of *TMEM37 *and *LASS5 *were not detected, although the sense transcripts of these two genes were expressed (Figure 5D), which was in consistence with the MPSS data. These interesting results, that the antisense transcripts of some genes were in fact co-expressed with the sense ones, suggested that the post-transcriptional regulation at mRNA level of some genes could be an important event through degenerating the sense RNAs or editing the mRNA for generating different splicing [18-20].

**Figure 6:**
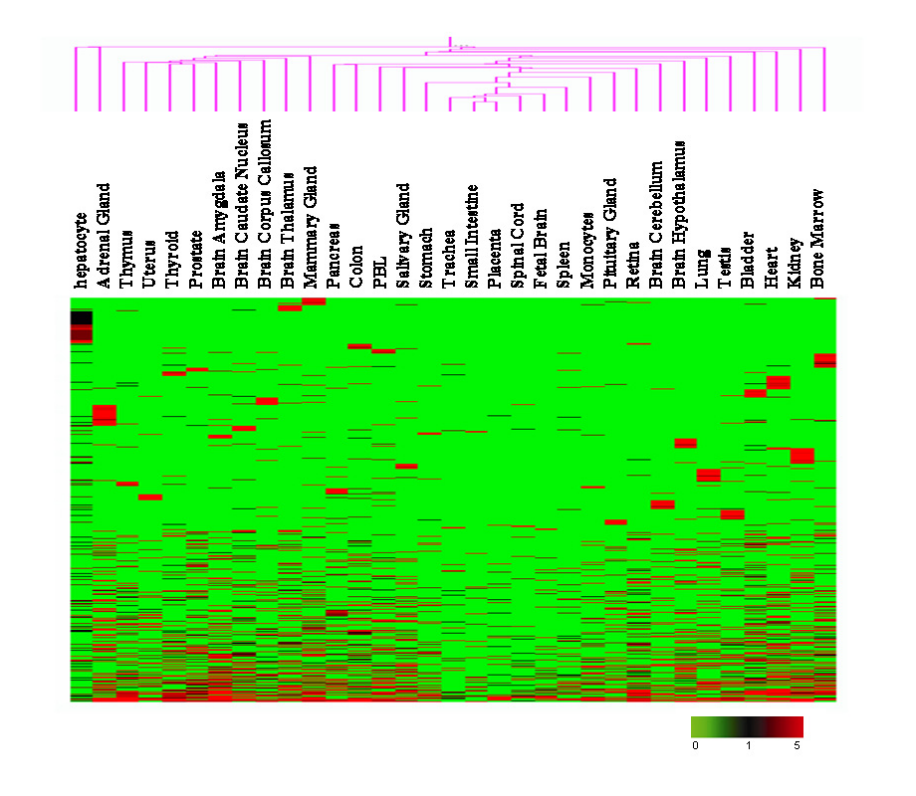
Hierarchical cluster analysis of the antisense transcripts from human tissues. The clustering was performed to analyze the expression pattern of 7830 antisense transcripts based on the MPSS signatures from 33 human tissues using a log_2 _pseudocolor scale, as indicated by fold ratios in the legend at the bottom. Green and black denote 0 and 1 TPM, respectively; whereas red indicates MPSS signatures with more than 1 TPM.

To further characterize the antisense transcripts' tissue enrichment, the NATs data from the hepatocytes was compared with that of published MPSS data from another 32 tissues [6]. Here, NATs were considered as tissue-enrichment where the S_value was > 2. Many NATs exhibited significant tissue-enriched expression patterns (Figure 6) (see additional file 5), suggesting that the expression of these NATs could be strictly regulated in a given tissue. Among 93 potential hepatocyte-enriched NATs (see additional file 6), some antisense transcripts may be involved in pentose and glucuronate interconversions (*DCXR*, *UGT2B4 *and *UDP*), fatty acid metabolism (*EHHADH*), bile acid biosynthesis (*BAAT*), urea cycle (*CPS1*), and arachidonic acid metabolism (*CYP2C8, CYP2C9, CYP2E1 *and *AKR1C3*).

**Figure 7:**
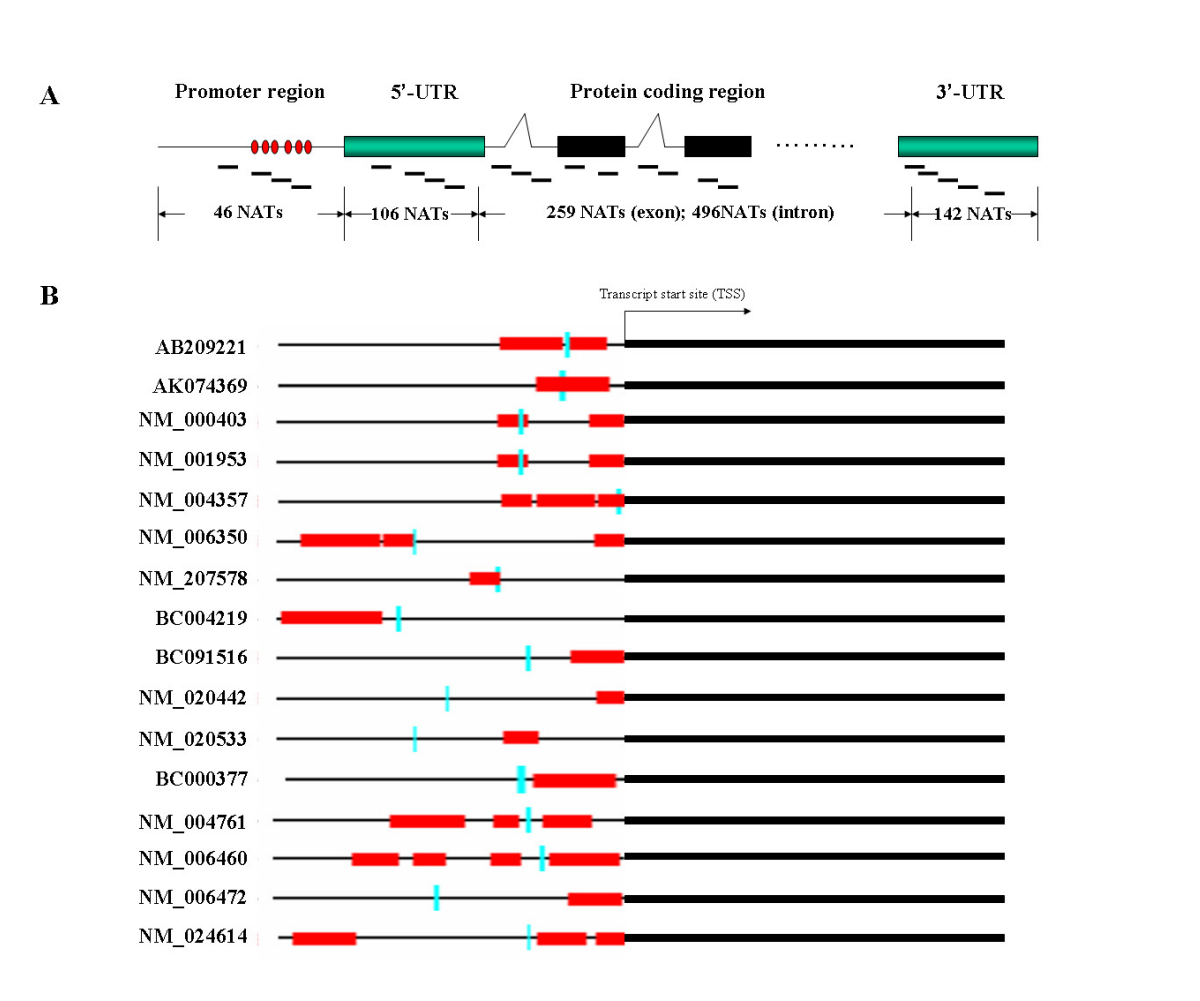
Genomic localization of antisense transcripts mapped onto gene structures. **A**. Distribution of 1049 antisense transcripts mapped onto the promoters, 5'-UTRs, protein-coding regions and 3'-UTR, respectively. Red cycles indicate the CpG islands of those promoters, and the green and black boxes represent the UTRs and exons, respectively. The short lines indicate the distribution of MPSS signatures of those antisense transcripts. **B**. Genomic localization of 16 NATs mapped onto the CpG-island-enriched promoters of the corresponding genes. The red boxes indicate CpG islands and the vertical lines in light green indicate the location of MPSS signatures.

To explore the genetic mechanisms involved in the regulation of these NATs, all NATs were mapped onto the genomic loci in order to investigate the genomic features of their corresponding sense transcripts. Among 1,819 NATs, 1,049 NATs were uniquely mapped onto genomic loci, where 507 (48.33%) were matched onto the exons of the corresponding genes, including 106 (10.11%), 142 (13.54%), and 259 (24.69%) assigned onto 5'-UTR, 3'-UTR, and protein-encoding regions, respectively, according to the structures of mRNAs derived from these genes (Figure 7A). Furthermore, additional 46 (4.38%) and 496 (47.28%) NATs were localized onto the promoters and intronic regions of genes, respectively, implying that the regulatory mechanisms of transcriptional expression involving antisense RNAs are very complex, although the possibility that these NATs are polyadneylated in the opposite direction of the sense strand transcripts does not exclude. Interestingly, of 46 NATs matched to the promoters, 16 could be transcribed from the CpG island-enriched promoters (Figure 7B, see additional file 7). Recently, some groups have reported that the association of antisense RNA transcripts with CpG methylation can lead to human genetic diseases [20,21]. Whether these NATs in hepatocytes could regulate the transcriptional efficiency of sense transcripts through the DNA methylation of the promoters should be further investigated.

To evaluate the evolutional conservation of these NATs, we identified their ortholog pairs between human and mouse through the reciprocal best matches between both genomes. 167 (10.40%) out of 1,605 human NATs were considered to have the corresponding antisense orthologs from mouse through comparing the data from the HomoloGene database with Mouse Genome Informatics, suggesting that some NATs could be evolutionarily conservative. Furthermore, these human NATs derived from hepatocytes were compared with 1,127 mouse NATs from the liver based on MPSS signatures deposited in NCBI GEO (GPL1010). The resulting data showed that 167 NATs, including 146 with both sense and antisense transcripts, were commonly expressed in human and mouse livers (see additional file 8). For example, the NATs of these genes, such as *CYP4A2, HADH2, HSD17B4 *and *ACOX2 *involved in fatty acid metabolism, *CYP27A1 *and *ACADSB *in bile acid biosynthesis, and *ADH4 *in alcohol metabolism, were conserved in both human and mouse livers, suggesting that these conservatively co-expressed sense-antisense pairs could play important roles in hepatic functions.

## Conclusion

This study provided a comprehensively transcriptomic atlas of human hepatocytes using MPSS technique, which could be served as an available resource for an in-depth understanding of human liver biology and diseases. In addition, the data suggested that, like a large number of protein-coding genes, some antisense transcripts expressed in hepatocytes could play important roles in transcriptional interference via *cis*-/*trans*-regulation mechanisms.

## Methods

### Adult human livers

Normal human livers from ten patients were resected surgically because of hemangioma in liver in China. The samples were obtained from the portion unaffected by the hemangioma and frozen in liquid nitrogen immediately. All procedures and risks were explained verbally and in a written consent form. The samples were sectioned and confirmed to be normal histologically. All laboratory data assessing hepatic function were within normal ranges, including serum alanine aminotransferase, aspartate aminotransferase, g-glutamyl transpeptidase, alkaline phosphatase, total bilirubin, albumin, prothrombin activity, glucose, cholesterol, and triglycerides (data are not shown). Serological tests for hepatitis B surface antigen, Hepatitis C virus antibodies, and Human immunodeficiency virus antibodies also showed negative. Neither heavy alcohol consumption nor the intake of chemical drugs was observed before surgical resection.

### Laser Capture Microdissection and RNA extraction

Four- micrometer sections of frozen liver tissues were not stained with any dye. The sections were immediately microdissected with a Leica AS LMD Laser Capture Microdissection System (Japan) using laser pulses of 7.5-μm diameters, 70 mW, and with 2–3 ms duration. Approximately 10^6 ^cells of human hepatocytes were microdissected and stored on microdissection caps with TRIZol reagent. Each cell population was determined to be 95% homogeneous by microscopic visualization of the captured cells. Laser-capture-microdissected hepatocytes were added to 1 ml TRIZol reagent (Invitrogen, Carlsbad, CA), and then total RNA was extracted according to the manufacturer's instructions and RNAse-free DNase I was used to remove DNA contamination. The nucleotide acid concentration and purity were assessed at 260 nm using a spectrophotometer (DU 530, Beckman-Coulter Inc., Fullerton, CA), and the quality was assessed by an Agilent 2100 Bioanalyzer. In addition, for the purposes of considering gene expression polymorphism, the total RNAs from ten livers were pooled equally.

### MPSS

MPSS was performed using RNA from the pooled livers and evaluated for the presence of LZP markers and absence of markers for AFP. The mRNA was converted to cDNA and digested with DpnII. The last DpnII site and the downstream 14 bases were cloned into Megaclone vectors and their sequences determined according to the MPSS protocol. This experiment was carried out by TaKaRa Co., Japan.

### Semi-quantitative Reverse Transcription PCR

Reverse Transcription (RT) was performed in a 20 μl reaction system which contained 2 μg total RNA, 20 pmol oligo-dT, mixed up to 11 μl with DEPC-H2O and then incubated at 70°C for 5 minutes. After 5 mintues at 0°C, 4 μl 5 × buffer, 2 μl 0.1 M DTT, 2 μl dNTP (10 Mm) and 1 μl (200U) SurperScript II reverse transcriptase (Life Technologies), incubated at 42°C for 2 hours. In PCR, β-actin was used as a control to estimate the quality of cDNA (forward primer: 5'-TCACCCACACTGTGCCCATCTACGA-3' and reverse primer: 5'-CAGCGGAACCGCTCATTGCCAATGG-3'). To further avoid DNA contamination, all primers in this study were designed to span at least one exon. Each PCR was performed as follows: pre-denature at 94°C, 5 min; denature at 94°C, annealing at 55°C, extend at 72°C, 40 seconds, respectively, and finally at 72°C for 7 min. The PCR products were observed by electrophoresis on 2% agarose gel.

### Data source

32 tissues for human transcriptomic analysis by MPSS were extracted from the NCBI GEO database (GPL1010). Genomic mapping data were taken from the human genome database version 17 at UCSC.

## List of abbreviations

MPSS: Massively Parallel Signature Sequencing

LCM: Laser Capture Microdissection

## Authors' contributions

JH and ZGH designed the research pipeline. YLZ, FXD, QD and WY carried out the PCR experiments. YH and HYW captured human hepatocytes using LCM technique, JH, PH, XWW, XGZ and TTL performed the statistic analysis. JH, PH and ZGH drafted the manuscript. YXL, PYY and HYW participated in the design of the study. ZGH conceived and oversaw the research. All the authors read and approved the final manuscript.

## Availability and requirements

All data is available by download from our website .

## Supplementary Material

Additional File 1 Classification of the MPSS cDNA signatures Click here for file

Additional File 2 List of UniGene clusters expressed in hepatocytes Click here for file

Additional File 3 List of 327 potential hepatocyte-enriched UniGene clusters Click here for file

Additional File 4 List of sense and antisense transcripts co-expressed in human hepatocytes Click here for file

Additional File 5 List of NATs in 33 tissues Click here for file

Additional File 6 List of antisense transcripts enriched in human hepatocytes Click here for file

Additional File 7 Genomic locations of the antisense transcripts on the promoter regions Click here for file

Additional File 8 List of both sense and antisense transcripts co-expressed in human hepatocytes and mouse liver Click here for file
